# Supplementary material for: Complexome profiling of the Chlamydomonas psb28 mutant reveals TEF5 as an early PSII assembly factor
Source: Plant Cell. 2025 Mar 17;37(6):koaf055. doi: 10.1093/plcell/koaf055 (PMC12164590; doi:10.1093/plcell/koaf055)
Supplement: koaf055_Supplementary_Data [file koaf055_supplementary_data.zip › psb28_interactive.zip!/plots/intensity/qualityPlots/peptides/_Cre06.g267300.t1.2.html]

### Cre06.g267300.t1.2

Trivial name:   
SeqCoverage [median]: 14.70  
Mass: 53.96 kDa  
  
 

| Sequence | MissedCleavage | Mass [Da] | wtIntensity | muIntensity | Score |
| --- | --- | --- | --- | --- | --- |
| AIGEGHMLVQGASR | 0 | 1424.72 | 1568.75 | 3980.56 | 79.88 |
| EADIGLAMAR | 0 | 1045.52 | 452.24 | 1715.66 | 103.83 |
| EVEGLGR | 0 | 758.39 | 0.00 | 1571.95 | 73.04 |
| SATQLLTASR | 0 | 1046.57 | 0.00 | 1337.17 | 80.69 |
| SPEQQAEALESLRK | 1 | 1584.81 | 604.39 | 1888.29 | 49.33 |
|  |  |  | 2625.4 | 10493.6 |  |
